# Mindfulness and inhibitory control: Insights from the stop signal task with neutral and reward-associated stimuli

**DOI:** 10.1371/journal.pone.0303384

**Published:** 2024-05-22

**Authors:** Zsófia Logemann-Molnár, Anna Veres-Székely, Zsolt Demetrovics, H. N. Alexander Logemann

**Affiliations:** 1 Doctoral School of Psychology, ELTE, Eötvös Loránd University, Budapest, Hungary; 2 Institute of Research on Adult Education and Knowledge Management, ELTE Eötvös Loránd University, Budapest, Hungary; 3 Institute of Psychology, ELTE, Eötvös Loránd University, Budapest, Hungary; 4 MTA-ELTE Lendület Adaptation Research Group, Institute of Psychology, ELTE Eötvös Loránd University, Budapest, Hungary; 5 Centre of Excellence in Responsible Gaming, University of Gibraltar, Gibraltar, Gibraltar; 6 College of Education, Psychology and Social Work, Flinders University, Adelaide, Australia; 7 Department of Clinical, Neuro and Developmental Psychology, Vrije Universiteit Amsterdam, Amsterdam, Netherlands; University of Technology Sydney, AUSTRALIA

## Abstract

Mindfulness has been linked to enhanced inhibitory control, yet the dynamics of this relationship, especially in reward situations, are not well understood. Our cross-sectional study aimed to explore the relationship between trait mindfulness and a performance measure of inhibitory control as a function of reward context operationalized by stimuli characteristics, and temporal inhibitory demands. Thirty-six individuals aged 19 to 41 filled out the Mindful Attention Awareness Scale (MAAS) and performed a stop signal task (SST), that included both neutral (stone images) and reward-related (money images) stimuli. The SST encompassed four conditions: neutral go/neutral stop, neutral go/reward stop, reward go/neutral stop, and reward go/reward stop, requiring participants to suppress reactions either during or after encountering reward-related stimuli. The relevant index of inhibitory control is the stop signal reaction time (SSRT), a performance measure of inhibitory control. Our findings showed no notable variation in the relationship between the MAAS score and SSRT across the different conditions. However, there was an overall significant effect of MAAS score on SSRT, irrespective of condition. Results may reflect the benefit of mindfulness on inhibitory control after generalized reward exposure.

## Introduction

Effective inhibitory control is crucial, especially in reward contexts, and it is known that poor inhibitory control plays an important role in various disorders. The connection between inhibitory control and mindfulness has garnered considerable attention in research. While a substantial body of literature supports the positive association between mindfulness and executive functions [1]. However, recent studies focusing specifically on inhibitory control suggest that the nature of this relationship may depend on the exact reward context and the timing of inhibitory control engagement—whether it occurs concurrently with exposure to reward-associated stimuli or subsequently [2, 3]. Considering these discrepancies, the primary objective of the present cross-sectional study was to elucidate the aforementioned ambiguity and provide a clearer understanding of the interplay between trait mindfulness and inhibitory control as indexed by performance as a function of reward context and inhibitory requirements.

Mindfulness can be described as the capability to consciously focus on both internal experiences and external occurrences in an open, discerning, and attentive way [4]. While the precise neurological underpinnings of mindfulness remain somewhat elusive, advancements in understanding are being made. Notably, as argued previously [2, 5], research reveals a substantial overlap with respect to the neuroanatomical regions that are thought to be associated with trait mindfulness and that of induced mindfulness [6, 7]. To elaborate, training in mindfulness has been shown to improve trait mindfulness [8, 9], with both forms of mindfulness linked to decreased activity in the Default Mode Network (DMN) regions [6, 7]. This complex network is thought to drive adaptive behaviour and self-initiated thought. Importantly, the DMN and Salience Network (SN) show a complex interaction, with mindfulness having varied impacts on each [6, 10]. The SN plays a central role in detecting and redirecting attention towards significant external stimuli. It has been observed that when the SN is activated, there is a corresponding reduction in DMN activity [11].

Mindfulness-based interventions have demonstrated favorable impacts on executive functions at the cognitive/behavioral level. Previous research indicates positive outcomes in terms of performance measures [1, 12–16] and corresponding electrophysiological responses influencing these performance benefits [12]. Inhibitory control, a crucial aspect of executive functions characterized by the ability to stop prepotent responses, is often assessed objectively through tasks such as go/no-go tasks [17] or a stop signal task [18–20]. While there are indications of specific positive effects on inhibitory control resulting from mindfulness, many studies have used measures that pose challenges in isolating inhibition-associated processes from other contributing factors to inhibition performance [1].

Given the aforementioned information, it may appear counterintuitive that previous research has indicated a negative correlation between mindfulness and right Inferior Frontal Gyrus (rIFG) activity [21], a region linked to inhibitory control [22]. However, the connection between mindfulness and inhibition could be contingent on the timing of the inhibitory demand. For example, within an attentional blink paradigm, studies have demonstrated that mindfulness is linked to enhanced disengagement of attention from previously highlighted stimuli [23]. This aligns with another investigation, revealing that participants who underwent brief mindfulness training exhibited reduced carryover effects from previous task sets in a series of experiments [24]. These findings hold significance for tasks that measure inhibitory performance where an inhibitory requirement typically follows a distinct task set, such as the need to respond rapidly and accurately to a stimulus requiring a response, and inhibiting in response to a stop signal [19]. It may be expected that, especially in neutral contexts, mindfulness facilitates inhibitory control in such tasks, notably because of reduced carry-over effects from the primary task (responding to the go stimuli) to the secondary task (withholding the prepotent response).

The previously mentioned findings concerning the link between mindfulness and inhibitory control specifically apply to neutral situations. However, it is crucial to recognize that individuals are not operating exclusively in neutral environments; they often find themselves in situations linked to rewards, such as encountering tempting food-related or money-associated stimuli. Notably, both palatable food [25, 26] and money cues [27] are known to activate brain circuitry responsible for processing reward-related information, particularly the striatum. This activation is linked to heightened approach tendencies toward such stimuli and presents inhibitory challenges [25, 26]. This emphasizes the necessity of considering the reward context when assessing the relationship between mindfulness and inhibition. Additionally, when considering the definition of mindfulness and its connection to reduced Default Mode Network (DMN) activity, along with the enhanced activity of components in the Salience Network [6, 10], it is conceivable that mindfulness is associated with attentional bias for stimuli that are associated with reward, which may challenge inhibition in response to such stimuli.

Studies at the behavioral and electrophysiological levels offer preliminary support for this notion. Specifically, research employing oddball paradigms indicates a potential link between increased mindfulness and heightened attentional capture of significant stimuli [28–30]. This suggests that heightened mindfulness might lead to more immediate attentional capture by salient stimuli, such as those related to rewards, potentially complicating inhibitory control when directly exposed to such stimuli. In addition, in a recent study that incorporated a go/no-go paradigm, inhibitory control was challenged in a context of reward- relative to neutral stimuli, especially for individuals scoring high on trait mindfulness [2]. Further support was evidenced in a previous study performed by Andreu et al. [31]. In this investigation, participants (smokers and non-smokers) were allocated to either a short mindfulness intervention or a control group. They participated in a go/no-go task featuring images related to smoking. The findings revealed that the stop-stimulus associated P300 was decreased after mindfulness training, suggesting a reduction in inhibitory activity [17, 31, 32].

However, given the previously mentioned mindfulness-associated reduced carry-over effects [24], results may differ when inhibition is required *subsequent* to being presented with reward-associated stimuli. Indeed, a recent study that incorporated a stop signal paradigm, with reward-associated stimuli (images of money) requiring a response, followed by neutral stop stimuli, showed that mindfulness was associated with improved inhibitory control in such context [3].

Taken together, it appears that there is a relationship between mindfulness and enhanced inhibitory control in neutral contexts. However, the relationship may vary in reward-associated contexts based on whether inhibitory control is necessitated during exposure to reward cues or after such exposure. Surprisingly, this critical question remains unexplored within a single experimental framework.

In our present investigation, we measured trait mindfulness and employed a stop signal task that yields a performance measure of inhibitory control. The task featured four conditions, wherein go and stop stimuli were either neutral (images of stones) or reward-associated (images of money). No actual rewards were provided, and the reward-associated stimuli were not contingent on performance. The following hypotheses were postulated. Firstly, a heightened challenge to inhibitory control in any context associated with rewards was expected. Secondly, we expected that mindfulness is associated with improved inhibitory control when inhibition was required following a neutral stimulus after exposure to a reward-associated stimulus, as compared to a neutral context. Thirdly, conversely, an inverse relationship was expected when inhibitory control would be required during immediate exposure to reward stimuli, irrespective of whether the preceding go stimulus was neutral or reward-associated.

## Materials and methods

### Sample

Participants were recruited through social media. To be eligible, participants had to be between 18 and 50 years old, have no neurological or psychological disorders, and not have used any drugs in the past 7 days. The sample size was determined using G*Power [33]. Assuming a medium effect size, power of .80, and alpha set to .05, a sample size of 28 or higher was required. Participants did not have to be native English speakers, but those who did not meet at least a B1 level of English, according to the Common European Framework of Reference for Languages—Self-assessment grid, were excluded. In addition, participants who had invalid (negative) SSRTs, an inhibit rate higher than .75 or below .25, an omission rate exceeding .1 and/or an incorrect rate to go stimuli exceeding .5, were also excluded [20]. In total, 48 participants were excluded due to these stringent exclusion criteria. The final sample consisted of 36 participants: 27 women and 9 men, aged between 19 and 41 years old (mean = 24, SD = 4). The average MAAS score was 3.90 (SD = .72) and ranged from 2.13 to 5.27. Participants provided written informed consent before the experimental procedures, and the study was approved by the Research Ethics Committee of the Institute of Psychology, Eötvös Loránd University (ELTE), and conducted in accordance with the Declaration of Helsinki. The recruitment period was from the 1^st^ of April 2022 until the 1^st^ of November 2023.

### Materials

#### Psytoolkit

Psytoolkit [34, 35] was used for online cognitive psychological experiments that included behavioral and self-report assessments. The tool is highly replicable, even compared to offline implementations [36, 37].

### Language proficiency according to the Common European Framework of Reference for Languages-Self-assessment grid

The language proficiency questionnaire had a single question that asked about the participant’s English reading proficiency. There were six response options to choose from based on the Common European Framework of Reference for Languages. These options ranged from "A1 Basic user" to "C2 proficient user." For instance, a sample response for the "B1 Independent user" level was "I can understand texts that primarily use high-frequency everyday or job-related language. I can also comprehend the description of events, feelings, and wishes in personal letters." This level of proficiency was the minimum requirement for the current research.

#### Mindful Attention Awareness Scale

Dispositional mindfulness was assessed using the 15-item Mindful Attention Awareness Scale (MAAS) [38]. An example item is, for instance, "I could be experiencing some emotion and not be conscious of it until some time later." Participants responded on a six-point scale ranging from "Almost always (score 1)" to "Almost never (score 6)." The MAAS score represents the average item score, with a higher score indicating a greater level of mindfulness. The scale is known to have a high reliability with Cronbach’s alpha exceeding .8 [38].

#### The stop signal task

**Fig 1:**
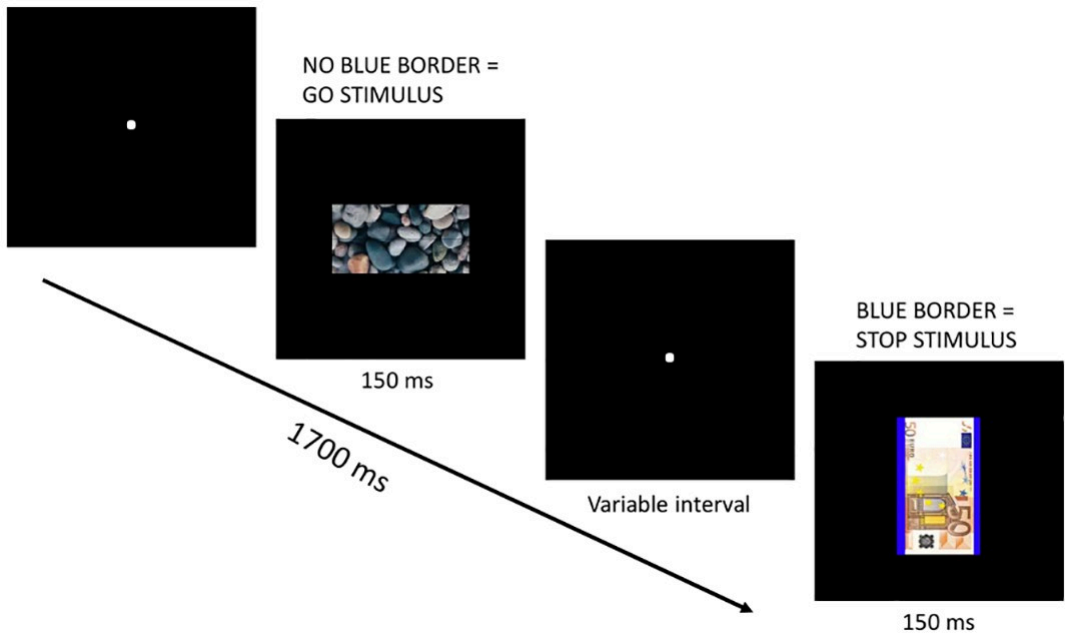
An example of a stop trial in the SST.

The online stop-signal task (SST) was implemented based on its original conceptualization [39], and was adapted from a previous implementation [3]. In the SST, participants had to respond to go stimuli by pressing either a left or right button using their index finger. Occasionally, the go stimuli were followed by stop stimuli, which required the participant to withhold their prepotent response. The stimuli were presented centrally, and the duration of each stimulus was fixed at 150ms. The intertrial interval was 1500 ms for go trials and 1700 ms for stop trials. Initially, the interval between the onset of the go stimulus and the stop stimulus was set at 250ms. This interval was then adjusted dynamically after each stop trial using a tracking algorithm. If a participant failed to inhibit their response, the stop-signal delay was decreased by 50ms. If a participant successfully inhibited their response, the stop-signal delay was increased by 50ms. This tracking algorithm ensured an approximate 50% inhibition rate, which increases the reliability of the estimation of the SSRT [19, 20]. The task included a practice condition and four experimental conditions: neutral go + neutral stop, neutral go + money stop, money go + neutrals stop, and money go + money stop. The practice block consisted of 48 go trials and 16 stop trials, with participants receiving immediate feedback for errors of omission, commission, and incorrect responses. Each experimental condition consisted of 96 go trials and 32 (25%) stop trials. The only difference between the experimental conditions was whether the go and stop stimuli represented stones (four types) or money (four types). The stop stimulus was identifiable by a small blue border of 15 pixels (see Fig 1 for an example trial). In all experimental conditions, the go and stop stimuli were randomly displayed in either portrait mode (go: 100x200 pixels; stop (including border): 100x230 pixels) or landscape mode (go: 200x100 pixels; stop (including border): 230x100 pixels). The required keyboard response to the go stimulus was determined by its orientation. Trials were randomized for each participant, and the condition order of the experimental conditions was counterbalanced across participants. The computation of the SSRTs aligned with the most recent consensus [20]. Specifically, we employed the integration method, and omissions were replaced with the maximum response time within the specified response window (1500ms). RTs that were either under 150 milliseconds or surpassed the duration between trials were excluded. For the go trials, the RTs were arranged in ascending order. The specific RT value used for calculating SSRT was found by identifying the position in this ordered list corresponding to the proportion of failed stop trials. SSRT was then computed by deducting the average time difference between go and stop signals from this identified RT.

### Procedures

The participants were recruited through social media. After reading the information letter and providing written informed consent, they started filling out the questionnaires. Following this, they were given instructions for the stop-signal task. Specifically, before each condition, participants were instructed to respond as fast and accurately as possible and to not wait for the stop signal. To ensure that participants understood the instructions, they were provided with feedback during the practice part of the task. Once the participants completed all the conditions, the experiment was concluded.

### Statistical analyses

To test our hypotheses, a repeated measures ANCOVA was performed with Mindfulness as between-subjects covariate, condition as within-subjects factor with four levels, and SSRT as main outcome variable. Alpha level was set at 0.05. For visualization purposes only, the continuous MAAS score was transformed to a categorical variable using median-split.

## Results

Descriptive SST performance data is shown in Table 1.

**Table 1:** Summary of performance data in the SST.

Condition	SSRT	Mean RT	STD RT	SOA	Prop Correct	Prop Omissions	Prop Inhibition
Neutral-Go + Neutral-Stop	227	743	190	497	0.95	0.02	0.52
Neutral-Go + Money-Stop	240	758	183	507	0.95	0.02	0.50
Money-Go + Neutral-Stop	229	759	201	516	0.94	0.03	0.50
Money-Go + Money-Stop	235	754	194	511	0.94	0.02	0.50

Note: This table presents a summary of reaction time (RT) and performance metrics across conditions. Stop Signal Reaction Time (SSRT), Mean RT, Standard Deviation of RT (STD RT), Go-stop Stimulus Onset Asynchrony (SOA), and proportions of correct responses, omissions, and inhibitions are reported. Values are rounded for clarity, with SSRT, Mean RT, STD RT, and SOA rounded to the nearest whole number, and proportions rounded to two decimal places.

### Primary analyses

**Fig 2:**
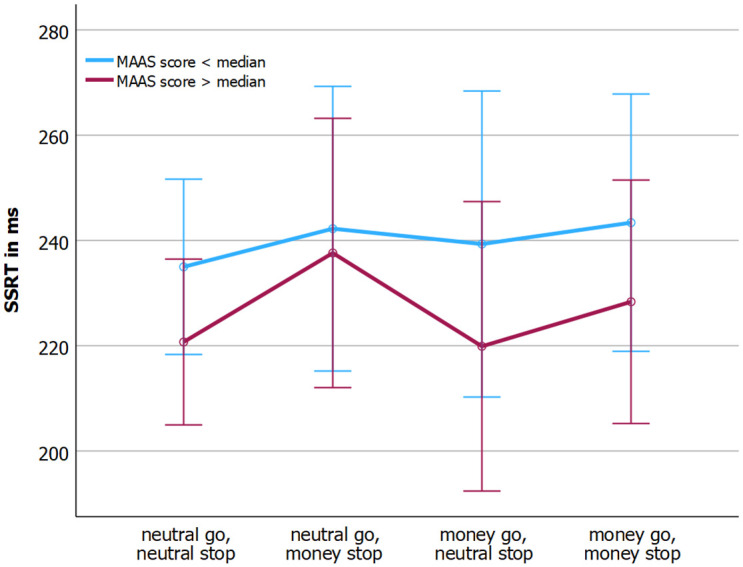
MAAS score and SSRT for each condition.

The relationship between MAAS score and SSRT was not different over conditions, F(3,102) = 0.18, p = .911, partial η^2^ = 0.005. Similarly, SSRT did did not differ across conditions, F(3,102) = 0.09, p = 0.966, partial η^2^ = 0.003. However, a significant main effect of MAAS score with respect to the SSRT was observed, F(1,34) = 5.27, p = 0.028, partial η^2^ = 0.134. For visualization purposes only, the MAAS variable was made categorical using median split, and this categorical score was plotted against SSRT (Fig 2). The figure shows that higher MAAS scores were associated with lower SSRTs, indicating better inhibitory control.

### Secondary analyses

**Fig 3:**
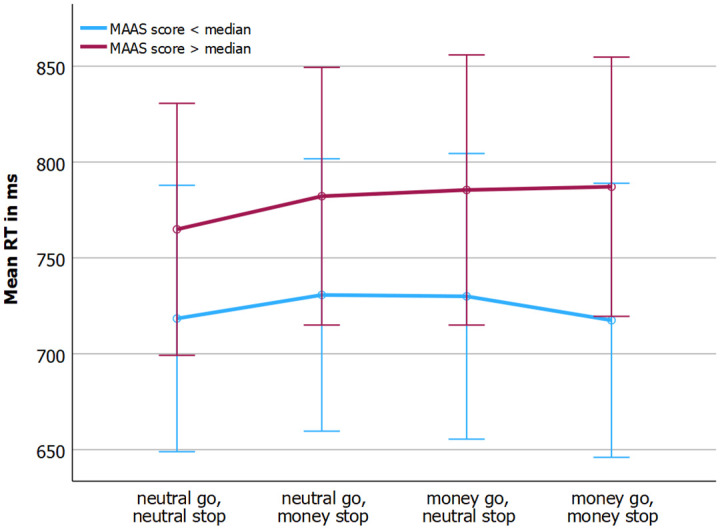
MAAS score and mean response time to go stimuli for each condition.

We also analyzed the effects on Mean response time (RT) to go stimuli (Fig 3). The sphericity assumption was violated, as evident by a significant Mauchly’s Test of Sphericity (p = 0.024). Hence, for the analyses regarding mean RT, Greenhouse-Geisser correction was employed. There was no relationship between MAAS score and mean RT as a function of condition, F(3, 80.67) = .54, p = 0.615, partial η^2^ = 0.016. Mean response time was not different across conditions, F(3, 2.37) = 0.38, p = 0.723, partial η^2^ = 0.011. There was a trend towards significance regarding the main effect of MAAS score regarding mean RT, with higher MAAS scores associated with slowing of responses, F(1,34) = 3.54, p = 0.069, partial η^2^ = 0.094. There was no significant difference between the mean response time in the first presented condition (M = 741ms, SD = 135) and the last presented condition (776ms, SD = 161), F(1,35) = 3.73, p = 0.061. Lastly, as evident from Table 1, the omission rate was low, but significantly differed from zero, F(1, 34) = 4.33, p = 0.045. There was no significant relationship between MAAS score and omission rate and the relationship did not depend on condition, F(1,34) = 0.67, p = 0.418, F(3,102) = 0.65, p = 0.587.

## Discussion

The aim of the current study was to explore the potential association between trait mindfulness and a performance measure of inhibitory control, particularly in the context of reward stimuli. The hypotheses were partially supported. We found an overall benefit of mindfulness regarding inhibitory control, which is supported by several previous studies. However, in contrast to our hypothesis, this effect did not differ across conditions.

In our study, a significant main effect of the Mindful Attention Awareness Scale (MAAS) score on stop signal reaction time (SSRT) was observed, independent of condition. This aligns with prior research, which highlights the beneficial impact of mindfulness on executive functions [1]. Participants with elevated mindfulness scores exhibited enhanced inhibitory control. Contrarily, we did not find differences in the mindfulness-inhibitory control relationship across contexts that differ concerning reward. This is in contrast with previous studies suggesting context dependency in this relationship. In two recent studies focusing on inhibitory control, the association between mindfulness and control in neutral contexts was ambiguous, yet in reward contexts, the relationship was more pronounced, with the effect’s direction varying based on the timing of inhibitory demands to reward stimuli [2, 3]. It may be argued that our experimental design, which included three reward conditions and one entirely neutral condition, might have led to the spillover of the reward context’s effect into the neutral condition. Supporting evidence for this notion comes from a study on mindfulness’s impact on inhibitory control in smokers, where reward and neutral stimuli were intermixed, and an effect of mindfulness was found on an electrophysiological measure which did not vary by reward context [31]. The absence of a significant main effect of condition on SSRT, contrary to a previous study using similar stimuli but fewer conditions [3], further endorses this notion.

The question arises as to why the observed mindfulness-inhibitory control relationship was positive in a general reward context, rather than negative or nullified, as reported elsewhere [2, 3]. Although SSRT means (Fig 2) suggest greater challenges in inhibitory control in conditions where the stop stimulus is reward-associated, particularly at higher mindfulness levels, this effect was not statistically significant. The overarching benefit of mindfulness for inhibitory control across conditions might stem from the prioritization of visual information distinguishing the stop stimulus from the go stimulus. This suggests a potential attentional bias towards the salient blue border of the (most relevant) stop stimulus, with relative suppression of reward-associated information. This facilitated control in such contexts could be explained by mindfulness-associated reduced carry-over effects [24], in this instance, from the response task to the inhibitory task. One way to address the potential for reward-associated effect spillover is by having participants perform only one condition, and varying conditions across participants and not including conditions as a within-subject factor.

In view of the mentioned overlap of the mechanism involved in induced and trait mindfulness, it may be tempting to generalize our results to those expected with induced mindfulness. However, as argued in our previous report, the effects of mindfulness training may be different from that of trait mindfulness as mindfulness training may involve more than just the facilitation of mindfulness [2]. In other words, inherent non-specific effects may contribute to the observed effects of mindfulness training.

Some concerns have been raised about the potential limitations of conducting experiments online, particularly in terms of accurately measuring reaction times compared to a controlled laboratory setting. However, these apprehensions have been largely addressed by recent research, which demonstrates that online experimental platforms can offer a level of validity and reliability on par with traditional lab environments [37, 40]. While it’s acknowledged that online settings might introduce distractions leading to random errors and increased response variability, it’s important to note that such increased variability would primarily result in a higher incidence of false-negative results due to diminished statistical power. To counteract this, we implemented stringent selection criteria, excluding participants who exhibited invalid SSRTs or a go error rate higher than 50%. This ensured the retention of participants who were consistently attentive to the task at hand [41]. We should note that this additional criterion did not result in a substantial reduction of the sample, as only two additional participants were excluded. Analysis of the remaining sample revealed a notably low rate of omissions in response to go stimuli and a high accuracy rate, as detailed in Table 1.

Moreover, the study employed an adaptive tracking algorithm designed to maintain a correct inhibition rate around 50%, enhancing the accuracy of the SSRT measurements [19, 20]. Notably, the average SSRT recorded in the standard neutral condition aligns closely with results from controlled laboratory settings [42]. While the response times were marginally longer (by about 100 ms) compared to those in lab settings, this can be attributed to the use of more complex stimuli in our study, rather than simple letters, a factor known to extend response times [43]. Nevertheless, this minor discrepancy is unlikely to compromise the validity of the SSRT. Participants were clearly instructed to prioritize both speed and accuracy in their responses. Had the participants significantly slowed their responses, the inhibit rate, even with the tracking algorithm, would likely have exceeded 50%. However, such a trend was not observed in our data. To further assess whether there was any substantial slowing as a function of time on task, we compared the mean response time to go stimuli in the last presented condition relative to the first presented condition for each participant. Overall, participants responded only slightly slower (35ms) in the last presented condition relative to the first condition, but this difference did not reach significance (*p* = 0.061).

Lastly, it has been argued that the SSRT may also be affected by failures to initiate the inhibitory response to stop signals during lapses of attention [44]. The commonly applied non-parametric integration approach to estimate the SSRT does not capture such lapses of attention [20, 45]. In that vein, observed improvements in inhibitory performance evidenced by shorter SSRTs might also be explained (at least in part) by reduced lapses of attention. More recently, parametric approaches have also been applied that can estimate the contribution of trigger failures, and may better capture individual differences [46]. However, such an approach requires substantially more trials which was not feasible in the current online implementation due to increased chance for attrition [20, 47]. Thus, as it stands, one may argue that mindfulness-associated reduced SSRT might be explained (at least in part) by reduced lapses of attention. We explored this option by assessing whether mindfulness was associated with lapses of attention as indexed by omission rate [48]. We did not observe significant effects of mindfulness regarding omissions in our paradigm. However, we should note that the omission rate was generally low, so room to detect an effect was limited. Hence, we cannot fully rule out a potential contribution of lapses of attention.

In conclusion, our study suggests that mindfulness is overall associated with improved inhibitory control, but that the effect might be restricted to a generalized reward-associated context. This finding further supports the beneficial effects of mindfulness on cognitive functions and offers potential implications for interventions aimed at inhibitory control. It is important to gain further insights into the exact processes that contribute to the observed effects, and the applicability of mindfulness training in applied contexts.

## Supporting information

S1 Checklist Human participants research checklist. (DOCX)
